# Postpartum Invasive Group A Streptococcal Disease in the Modern Era

**DOI:** 10.1155/2008/796892

**Published:** 2008-12-31

**Authors:** David M. Aronoff, Zuber D. Mulla

**Affiliations:** ^1^Division of Infectious Diseases, Department of Internal Medicine, University of Michigan Health System, Ann Arbor, Michigan, MI 48109, USA; ^2^Department of Obstetrics and Gynecology, Paul L. Foster School of Medicine, Texas Tech University Health Sciences Center, 4800 Alberta Avenue, El Paso, TX 79905, USA; ^3^Department of Epidemiology and Biostatistics, University of South Florida College of Public Health, Tampa, FL 33612, USA

## Abstract

To describe the clinical features of individuals hospitalized for postpartum invasive group A *Streptococcus* (GAS) infection, a retrospective, population-based study of hospitalized patients in the state of Florida was conducted. Cases of postpartum invasive GAS infection (occurring within 42 days of delivery) were compared to women with other manifestations of invasive GAS disease with respect to their age at the time of admission. Four cases of postpartum invasive GAS infection were detected in this population, yielding a prevalence of 1.6% (4/257) of postpartum disease in this invasive GAS infection database. Patients presented a median of 4 days (mean of 9 days) after delivery with signs and symptoms of infection. Three cases were complicated by bacteremia and one patient had streptococcal toxic shock syndrome. Each patient received multiple antibiotics and survived. No patients received intravenous immunoglobulin. For comparison, a secondary retrospective investigation of a large hospital discharge dataset obtained from the Florida Agency for Health Care Administration was assessed for patients with puerperal GAS infections. This method yielded an additional three cases, whose clinical and demographic characteristics were summarized. These data highlight that postpartum invasive GAS infection continues to complicate pregnancy, though the frequency has decreased markedly over the past century.

## 1. INTRODUCTION

Puerperal sepsis causes at least 75 000 maternal deaths annually, mostly in developing countries, and the morbidity of puerperal infections affects an estimated 5%–10% of pregnant women [1]. At one time, group A *β*-hemolytic *Streptococcus* (GAS, *S. pyogenes*) was the major microbial cause of postpartum infection [2]. Indeed, in the late 19th century, Louis Pasteur is credited with discovering that puerperal sepsis was predominantly caused by streptococci [2]. While today other microbes such as group B streptococci, gonococci, *Chlamydia*, herpes simplex, genital *Mycoplasma*, and bacterial vaginosis are more often implicated in postpartum fever, severe cases of puerperal fever are almost always caused by GAS [1]. Worldwide, it is estimated that severe GAS disease (including acute rheumatic fever and invasive infections) is responsible for over 500 000 deaths annually [3]. 

It has been noted that the obstetric patient is especially vulnerable to invasive GAS infection acquired via disrupted mucosal or cutaneous barriers during delivery [4]. Postpartum GAS infections in the modern era are a mixture of illnesses of endogenous (patient) origin and acquired (iatrogenic/nosocomial) infections [4]. Outbreaks of postpartum GAS infection continue to be reported, and are often related to the spread of GAS among postpartum patients by healthcare works that are asymptomatic carriers [4].

The Centers for Disease Control and Prevention (CDC) conduct population-based, active laboratory surveillance for invasive GAS disease in the United States [5]. According to the CDC, approximately 232 cases of postpartum invasive GAS infection occurred in the United States in 1997, an incidence of 0.06 cases per 1000 live births [4]. We previously published an extensive investigation of invasive GAS infections that were reported to the Florida Department of Health between 1996 and 2000 [6]. The database used for our original study included 257 hospitalized patients, providing a relatively large sample size for exploring postpartum GAS infection. Therefore, the objective of this study was to report the detailed clinical and epidemiologic features of women hospitalized for postpartum invasive GAS disease in Florida during the four-year period of 1996–2000.

## 2. MATERIAL AND METHODS

### 2.1. Source of patients and inclusion criteria for primary retrospective study

Previously unpublished demographic and clinical data on postpartum invasive GAS disease from a study conducted in Florida, the methods of which are described in detail elsewhere, were accessed [6, 7]. The original study population was comprised of 257 patients who were hospitalized throughout the state of Florida between August 1996 and August 2000 for invasive GAS disease and reported to the Florida Department of Health (Tallahassee, Fla, USA). Invasive GAS disease was defined as isolation of group A *Streptococcus* from a normally sterile site (e.g., blood, cerebrospinal fluid, joint fluid, pleural fluid, or pericardial fluid) and a clinically compatible presentation. The definition of a clinically compatible presentation was one of several entities, including pneumonia, bacteremia in association with cutaneous infection (e.g., cellulitis, erysipelas, or infection of a surgical or nonsurgical wound), deep soft-tissue infection (e.g., myositis), meningitis, peritonitis, osteomyelitis, septic arthritis, postpartum sepsis (i.e., puerperal fever), neonatal sepsis, and nonfocal bacteremia. The original study also included cases of necrotizing fasciitis if GAS was isolated from a nonsterile site. Streptococcal toxic shock syndrome (STSS) was defined as reported by the CDC Working Group on Severe Streptococcal Infections [8].

For each case of invasive GAS disease that was reported by a county health department to the Florida Department of Health, a three-page case report form was completed after the review of the patient's medical record [7]. This chart review was usually performed by an epidemiology staff member at the reporting county health department. The surveillance case report form contained a section which required the chart reviewer to check off the diseases caused by group A *Streptococcus* including “endometritis/postpartum sepsis.” [9]. Another section captured information on underlying illnesses and risk factors including “pregnancy/peripartum” via a check box [9]. Cases were included in our current analysis if endometritis/postpartum sepsis was noted on the surveillance form and/or “pregnancy/peripartum” was checked off in the risk factor portion of the form and the infection occurred within 42 days of delivery. Although puerperal infection is most commonly encountered within the first 2 weeks after delivery, the definition extends to 42 days postpartum [1]. A cutoff of 42 days was employed to include the conventional duration of the puerperium, that period between the completion of delivery and the return of the reproductive tract to its nonpregnant condition. In addition, postpartum invasive GAS infections have been reported to occur up to 6 weeks after delivery [10]. The risk factor section included various conditions including obesity, diabetes, chronic lung disease, chronic heart disease, alcohol abuse, and noncutaneous malignancies. Antimicrobial therapy that was received during the hospitalization was also noted on the surveillance form.

### 2.2. Secondary comparative analysis

The health department surveillance dataset did not contain the patients' lengths of stay. Length of stay is a recognized measure of disease severity when assessing pregnancy-related morbidity [11]. Lengths of stay were available in a large Florida hospital discharge dataset that was obtained from the Florida Agency for Health Care Administration. As an alternative method and comparator to the above investigation, we retrospectively queried this Florida hospital discharge dataset. This public-use database includes discharge summaries from all nonfederal Florida hospitals except state tuberculosis and state mental health hospitals [12]. After data are entered into this system, they are subjected to formatting and logic checks. This dataset contained clinical and demographic information for 2, 343, and 330 patients who were hospitalized in a reporting facility and discharged in calendar year 2001. The principal discharge diagnosis and up to nine secondary discharge diagnoses were coded using the *International Classification of Diseases, Ninth Revision, Clinical Modification* (ICD-9-CM). Procedures performed during the hospital stay were also coded using ICD-9-CM.

The discharge database was searched for women carrying the diagnosis of postpartum invasive GAS disease who were residents of Florida. While there is no ICD-9-CM code for “invasive group A streptococcal infection,” postpartum, or otherwise, these cases can be found by searching for women whose electronic discharge record contained both an ICD-9-CM code for “major puerperal infection” (670.00, 670.02, and 670.04) and the code for “group A streptococcal infection” (041.01). Three such cases were found in the discharge dataset, and their clinical and demographic characteristics were summarized. Finally, the 2001 incidence rate of postpartum invasive GAS disease was calculated by dividing the 3 cases by the number of resident live births in Florida in 2001 [13].

### 2.3. Statistical analysis

SAS for Windows 9.1.3 (Cary, NC, USA) was used to manage and analyze the data. Cases of postpartum invasive GAS infection were compared to women with other manifestations of invasive GAS disease with respect to their age at the time of admission. Median ages are reported along with a two-sided *P*-value from a two-sample Wilcoxon test. The small number of postpartum cases (*n* = 4) prevented a comprehensive statistical analysis. Analyses of all databases were approved by the Institutional Review Board of the Texas Tech University Health Sciences Center School of Medicine at El Paso.

## 3. RESULTS

Clinical, laboratory, and epidemiologic features of the four cases identified by the Florida Department of Health are shown in Table 1. According to the public health surveillance form, none of the cases had pharyngitis, necrotizing fasciitis, or toxic shock syndrome caused by group A *Streptococcus*. The median age of the postpartum cases was 27.5 years while the median age of the 116 women hospitalized with other manifestations of invasive GAS disease was 54 years (two-sided Wilcoxon *P* = .04). GAS bacteremia was noted in cases 2 through 4 (Table 1). The hospital mortality rate was 0%. Of the three cases (cases 2 through 4) whose surveillance forms had known values for the receipt of IVIG, none had received IVIG during the hospital stay (data not shown). Patients presented a median of 4 days (mean of 9 days) after delivery with signs and symptoms of infection. One patient met the case definition of STSS (Case 3 below).

Case 1 was a 36-year old injecting drug user (Table 1) and had been a smoker in the 6 months preceding the current admission (data not shown). She developed peritonitis approximately 3 weeks postpartum with GAS. The interval between the onset of her symptoms and hospital admission was one day. The patient was admitted to the hospital from her doctor's office with the suspected (admitting) diagnosis of “rule out appendicitis.” She did not undergo surgery but was treated with parenteral cefazolin and metronidazole. Her highest temperature with 48 hours of admission was 103.0° F.

Case 2 was delivered her baby one day preceding the onset of GAS infection. Her surveillance form indicated the presence of pneumonia and GAS bacteremia secondary to the pulmonary focus of infection. She had cough, fever, vomiting, abdominal pain, and diarrhea at the time of (or within 48 hours of) admission. Her nadir blood pressure was 98/48 mmHg. The patient received doxycycline, vancomycin, gentamicin, metronidazole, and ampicillin/sulbactam.

Case 3 was admitted on the day that she developed symptoms of invasive GAS infection. She was a Hispanic woman admitted with postpartum endometritis and sepsis. She had undergone a bilateral tubal ligation at the time of her delivery, three days prior to her admission. Her case was complicated by STSS, as evidenced by hypotension requiring vasopressive agents (nadir systolic blood pressure on admission was 76 mmHg); hypoalbuminemia; a macular erythematous rash involving her abdomen and thighs; and elevated aspartate aminotransferase and alanine aminotransferase values on admission (609 U/L and 80 U/L, resp.) [14]. She was treated in an intensive care unit with gentamicin, clindamycin, penicillin, and cefepime and survived.

Case 4 was admitted with a diagnosis of postpartum fever two days after her symptoms began, though the onset of her infection in relation to her delivery date could not be determined. She was diagnosed with “bacteremia” and “endometritis/postpartum sepsis” on the surveillance form and GAS was isolated both from vaginal and blood cultures. Her highest temperature within 48 hours of admission was 101° F. She was treated with tobramycin, clindamycin, and ampicillin. She was discharged on hospital day five with a 10-day course of oral amoxicillin-clavulinic acid.

The demographic and clinical features of the three women hospitalized for a postpartum invasive GAS infection (who were identified through analysis of the Florida Agency for Health Care Administration discharge database) are shown in Table 2. Cases A and B appeared to have GAS bacteremia and sepsis, while cases B and C appeared to have GAS endometritis or a similar inflammatory disease of the uterus excluding the cervix (ICD-9-CM code 615.9). This code is used for the following diseases: endometritis, endomyometritis, metritis, myometritis, perimetritis, pyometra, or uterine abscess. All three cases survived and were discharged to their homes. The median length of stay was 3 days. Dividing the frequency of three cases by 205 800 live births yielded an annual incidence of 1.5 per 100 000 live births.

## 4. CONCLUSIONS

The objective of this analysis was to describe the clinical, laboratory, and epidemiologic features of women hospitalized for postpartum invasive GAS disease, an uncommon phenomenon. Using data from an active surveillance program that operated in 9 regions of the United States, the CDC calculated that 2.2% of all cases of invasive GAS disease are postpartum infections [4]. We report a similar, though somewhat lower prevalence: 1.6% (4/257) of the patients in our invasive GAS infections database were women who had postpartum disease.

Wahl et al. recently reported on 475 culture-confirmed cases of invasive GAS disease that were identified using a voluntary laboratory surveillance system in Germany [15]. Nine of these infections (1.9%) were related to pregnancy. Five of the cases delivered naturally while the remaining four women underwent a cesarean section. A limitation of the current study is that the mode of delivery was not recorded on the case report forms.

These four cases emphasize that invasive GAS infections occurring during the puerperium are not always limited to the reproductive tract. One mother in our study (case 1) was an injection drug user with peritonitis occurring approximately 3 weeks postpartum. Our research methods precluded an accurate determination of the timing of injection drug usage with the onset of peritonitis. However, intravenous drug use is a well-described risk factor for invasive GAS infection and GAS peritonitis has been reported in this population [16]. This case highlights the importance for clinicians to consider GAS infections in febrile or septic postpartum patients with known risk factors for this disease, such as diabetes, alcohol dependence, or injection drug usage. Another patient in our report (case 2) suffered from pneumonia. Though rare in the postpartum period, invasive GAS lower respiratory tract infections have been reported [4]. We cannot determine the pathogenesis of the pneumonia in our second case, which might have occurred as a complication of hematogenous spread from the reproductive tract, autoinoculation of the respiratory tract, or acquisition from another person.

One of the patients in our case series (case 3) had taken one or more nonsteroidal anti-inflammatory drugs (NSAIDs) during the seven days prior to the onset of GAS disease. It has been suggested that the use of NSAIDs might increase the risk of developing GAS necrotizing fasciitis or could facilitate the progression of invasive GAS disease to shock and multiorgan failure [17–19]. However, determining a causal association between NSAID use and infectious diseases has been difficult, particularly with retrospective studies [20, 21]. 

Our health department dataset (Table 1) spanning four years (August 1996–August 2000) captured one case of postpartum invasive GAS disease per year. To complement these data, we searched a Florida hospital database for women discharged throughout the state in 2001 whose records contained ICD-9-CM codes indicating the presence of postpartum invasive GAS disease. This is a logical source of supplemental epidemiologic information given the fact that a national study reported that 99% of women with postpartum invasive GAS disease are hospitalized for their illness [4]. The results from the health department database and the discharge database complement one another and are consistent with the epidemiology of postpartum invasive GAS disease. Both databases revealed that the majority of these patients do not have chronic comorbidities. Furthermore, all seven patients survived (Tables 1 and 2). According to CDC surveillance data, the case-fatality rate of postpartum invasive GAS disease is lower than the case-fatality rate of other invasive GAS infections in females of reproductive age (3.5% versus 9.4%) [4].

Obstetricians should be aware of the clinical features of GAS endometritis and/or disseminated infection. A review of 47 cases of intrapartum or postpartum GAS infection collected relevant clinical aspects of this disease [10]. The majority of patients (83%) presented within 7 days of delivery, though a minority, presented almost 8 weeks postpartum. Fever, usually of sudden onset at the time of presentation (or shortly thereafter), was nearly uniform. For 65% of patients, fever was the sole presenting symptom, while for others abdominal pain and/or heavy vaginal bleeding were significant complaints. A minority of women (three of the 47) developed septic shock [10]. It has been noted that women with postpartum GAS sepsis generally present within the first 24 hours after delivery with fever, hypotension, tachycardia, leukocytosis, hemolysis, and disseminated intravascular coagulation [22]. Such women can present without evidence for pelvic infection [23].

The medical management of invasive GAS infections is an evolving practice [17]. The CDC recommend high-dose parenteral penicillin and clindamycin for toxic shock syndrome or necrotizing fasciitis [24], a recommendation echoed by the Infectious Diseases Society of America [25]. The CDC also recommends for patients with severe illness, supportive care in an intensive care unit might be needed and early and aggressive surgery is often needed for necrotizing fasciitis cases to remove damaged tissue and stop disease spread [24].

The patients in our study were more often than not treated with multiple antibiotics. Two postpartum patients received clindamycin, which has been associated with a survival advantage over *β*-lactam antibiotics in some studies of invasive GAS [6, 26]. The mechanisms conferring a potential treatment advantage for clindamycin are complex (reviewed extensively in [14]). Clindamycin suppresses the production of GAS virulence proteins, enhances the phagocytosis of GAS by neutrophils, modifies host inflammatory responses, and is immune to the inoculum (“Eagle”) effect wherein GAS grown to high density or stationary phase exhibit decreased sensitivity to penicillins [17]. Clindamycin use has been advocated in the setting of STSS [27] and it is note worthy in this regard that case 3 in this report, who suffered from STSS, received clindamycin in combination with other antimicrobials and survived. This case also underscores the acute and fulminate nature that invasive GAS infections can assume.

The use of newer anti-Gram positive antibiotics such as dalbavancin, daptomycin, linezolid, and tigecycline in the setting of GAS infections remains to be defined [28]. If necrotizing fasciitis is suspected then rapid consultations with infectious disease clinicians and surgeons are imperative. The use of IVIG has been advocated for invasive GAS infections complicated by STSS (reviewed in [14]). None of our 4 postpartum patients received IVIG and this might reflect the low prevalence of STSS on clinical presentation.

Postpartum GAS infections can occur as a result of an outbreak on a maternity ward [29]. We did not have evidence that these four cases were related to an outbreak of invasive GAS infections, although our study was not designed to examine the interrelationships of cases in terms of chronology and location. The four cases reported here each occurred in a different calendar year, suggesting they were not part of one outbreak. Cases 1 and 2 resided in separate counties that do not border one another. Cases 3 and 4 were residents of the same county at the time of their illness; however, they were admitted almost 22 months apart indicating that a cluster in space and time was not present.

Although this study was limited by its retrospective nature, it confirms the uncommon occurrence of postpartum GAS infections in the modern era of antisepsis and antimicrobials. It also underscores the importance of recognizing GAS infection in the postpartum period and instituting antimicrobial therapy early in the course of infection to reduce the risk of an adverse outcome.

## Figures and Tables

**Table 1:** Clinical, laboratory, and epidemiologic characteristics of postpartum invasive group A streptococcal infections.

Case	Year of admission	Age (years)	Symptom onset (days postpartum)	Race and Hispanic ethnicity	Clinical presentation	Potential risk factors other than peripartum period	GAS* isolate site(s)	Outcome
1	1996	36	22	White non-Hispanic	Peritonitis	Injecting drug user	Peritoneal fluid	Survived

2	1997	26	1	Black non-Hispanic	Pneumonia	(None)	Genitals, blood	Survived
					Secondary bacteremia			

3	1998	29	4	White Hispanic	Endometritis/postpartum sepsis	Surgical wound	Blood	Survived
					Secondary bacteremia	Use of NSAIDs** in 7 days prior to onset of GAS illness		
					STSS^*#*^			

4	1999	25	NA	White non-Hispanic	Endometritis/postpartum sepsis	(None)	Blood, vagina	Survived
					Secondary bacteremia			

*Group A *Streptococcus*.

^†^At the time of admission.

^‡^NA: not available.

^*#*^STSS: streptococcal toxic shock syndrome.

**NSAIDs: nonsteroidal anti-inflammatory drugs.

**Table 2:** Clinical and demographic characteristics of women discharged in 2001 in Florida with ICD-9-CM codes* indicating the occurrence of a postpartum invasive group A streptococcal infection.

Case	Age (years)	Race and Hispanic ethnicity	Type of admission	Principal diagnosis	Selected secondary diagnoses	Principal procedure	Length of stay (days)	Outcome
A	36	Black Non-Hispanic	Emergent	Streptococcal septicemia	Major puerperal infection Acute posthemorrhagic anemia Pneumonia, organism unspecified Group A streptococcal infection Pain in cervical spine or neck Joint pain (pelvic region and thigh)	Venous catheterization not elsewhere classified (excluding that for cardiac catheterization or renal dialysis)	9	Survived

B	25	White Non-Hispanic	Emergent	Major puerperal infection	Streptococcal septicemia Retained portions of placenta or membranes, without hemorrhage Unspecified inflammatory disease of uterus—except cervix (e.g., endometritis) Group A streptococcal infection	Aspiration curettage of uterus	3	Survived

C	19	White Non-Hispanic	Emergent	Major puerperal infection	Unspecified inflammatory disease of uterus—except cervix (e.g., endometritis) Group A streptococcal infection	(None listed)	3	Survived

*ICD-9-CM: International Classification of Diseases, Ninth Revision, Clinical Modification.
